# The Role of Elderberry Hydrolate as a Therapeutic Agent in Palliative Care

**DOI:** 10.3390/antiox14020233

**Published:** 2025-02-18

**Authors:** Sara Gonçalves, Ana Caramelo

**Affiliations:** 1 Academic Clinical Center of Trás-os-Montes and Alto Douro (CACTMAD), University of Trás-os-Montes and Alto Douro, 5000-801 Vila Real, Portugal; caramelo.ana@utad.pt; 2 Department of Nursing, School of Health, University of Trás-os-Montes and Alto Douro, 5000-801 Vila Real, Portugal; 3 RISE-Health Research Network, Faculty of Medicine, University of Porto, 4200-319 Porto, Portugal

**Keywords:** elderberry hydrolate, palliative care, medicinal plants, antioxidant, quality of life, skincare

## Abstract

Elderberry hydrolate, derived from the berries of *Sambucus nigra*, has gained attention for its therapeutic properties, particularly in skincare. This review explores its potential applications in palliative care, where patients often experience compromised skin health due to illness or treatment. The bioactive compounds in elderberry hydrolate, including phenylacetaldehyde, 2-acetyl-pyrrole, n-hexanal, furfural, and (*E*)-beta-damascenone, contribute to its anti-inflammatory, antioxidant, antimicrobial, and skin-healing effects. These properties make it a promising option for addressing common dermatological issues in palliative care, such as irritation, dryness, pruritus, and inflammation. For example, phenylacetaldehyde’s antimicrobial and anti-inflammatory actions help soothe irritated skin, while 2-acetyl-pyrrole’s antioxidant effects protect sensitive skin from oxidative stress. Additionally, n-hexanal’s antimicrobial properties reduce infection risks and furfural aids in skin regeneration. (*E*)-beta-damascenone’s antioxidant effects help maintain skin health and prevent further damage. Despite these promising effects, barriers to the widespread implementation of elderberry hydrolate in palliative care exist, including cost, accessibility, patient sensitivities, and regulatory challenges. Future research focusing on standardized chemical profiling, clinical trials, and addressing these practical concerns will be crucial for integrating elderberry hydrolate into palliative care regimens. This review highlights its potential as a natural, supportive therapy for enhancing patient comfort and quality of life in palliative care settings.

## 1. Introduction

Palliative care is an essential aspect of healthcare focusing on improving the quality of life for patients with severe, life-limiting conditions [1]. It aims to alleviate suffering by addressing physical symptoms, such as pain, fatigue, and nausea, and psychological, social, and spiritual aspects of care. Among the various symptoms that patients in palliative care experience, dermatological conditions, including dry skin, irritation, pruritus, and inflammation, are common and can significantly affect patient comfort and emotional well-being [2]. These symptoms often go untreated or inadequately managed, leading to discomfort, decreased mobility, and reduced dignity and self-esteem. As a result, there is an increasing interest in exploring complementary therapies that can offer relief from such conditions, helping to improve patients’ overall experience during their final stages of life [3].

Currently, skincare in palliative care often relies on commercially available emollients, barrier creams, and wound dressings designed to manage dryness, irritation, and inflammation [4,5]. While these products are effective, there is growing interest in plant-derived alternatives that may offer additional bioactive benefits. Unlike conventional moisturizers, hydrolates provide a lightweight, water-based alternative with bioactive compounds that may contribute to skin healing, antimicrobial effects, and oxidative stress reduction [6,7].

One such therapeutic approach involves medicinal plants, which have long been a part of traditional healing practices due to their healing and soothing properties [8,9]. Among these, elderberry (*Sambucus nigra*) stands out as a plant of particular interest. Elderberry has been historically used for its medicinal properties, including its ability to support immune function, reduce inflammation, and promote skin health [10]. Its bioactive compounds, including antioxidants, flavonoids, and phenolic acids, have been recognized for their ability to combat oxidative stress, which often contributes to skin damage and irritation [10,11].

Elderberry (*Sambucus nigra*) has long been recognized for its medicinal and therapeutic properties, with its various forms, including extracts, tinctures, and hydrolates, being used in traditional medicine [12].

### 1.1. Origin and Distribution of Elderberry

Elderberry is native to Europe and has been cultivated and used for centuries for medicinal and culinary purposes [13]. The elderberry tree, *Sambucus nigra*, belongs to the family Adoxaceae and is widely distributed throughout temperate regions of Europe, Asia, and North America [14]. The plant grows in various environments, including woodlands, hedgerows, and along riverbanks, thriving in well-drained soils and temperate climates. Traditionally, the flowers and berries of the elderberry plant have been harvested for their medicinal properties, with the flowers being most commonly used for their soothing, anti-inflammatory effects. Elderberry is also cultivated for its decorative value and the health benefits attributed to its fruits and flowers [10,11].

### 1.2. Therapeutic Properties of Elderberry

Elderberries, specifically from the *Sambucus nigra* plant, have long been valued in traditional medicine for their wide-ranging therapeutic properties. The flowers, berries, and even the leaves of the elderberry plant have been used for centuries to treat various ailments, thanks to their rich composition of bioactive compounds, including antioxidants, vitamins, and flavonoids [15,16]. Recent research continues to confirm the beneficial effects of elderberries, particularly their role in supporting the immune system, reducing inflammation, and promoting skin health, all of which are valuable in palliative care settings [17,18].

#### 1.2.1. Antigenotoxic Properties

Elderberries have demonstrated significant antigenotoxic properties, which can help reduce or prevent genetic damage caused by harmful agents, such as radiation, toxins, or oxidative stress [19,20]. These effects are primarily attributed to their high content of anthocyanins and other phenolic compounds, which help neutralize reactive oxygen species and protect DNA from oxidative damage. Studies suggest elderberry extracts can reduce DNA strand breaks and improve cellular resilience against genotoxic agents. This property is particularly relevant in palliative care, where patients undergoing treatments like chemotherapy or radiation therapy are at risk of experiencing genetic damage that can lead to further health complications. By mitigating these effects, elderberries may help protect the integrity of healthy cells and reduce the side effects associated with such treatments.

#### 1.2.2. Immune Support and Antiviral Properties

Elderberries are most commonly known for their immune-boosting effects. The berries, in particular, contain high levels of flavonoids, such as anthocyanins, which have been shown to help modulate the immune system [15]. These compounds can enhance the body’s natural defense mechanisms by stimulating the production of cytokines, proteins that help fight off infections [11]. In addition to their immune-stimulating properties, elderberries have demonstrated antiviral effects, particularly against the influenza virus, by inhibiting the ability of the virus to spread within the body [21]. This makes elderberries a popular choice in traditional medicine, especially during cold and flu season, and could benefit palliative care patients more susceptible to infections due to weakened immune systems.

#### 1.2.3. Anti-Inflammatory and Antioxidant Effects

Elderberries are rich in antioxidants, including flavonoids and phenolic compounds, which help reduce oxidative stress in the body [10]. Oxidative stress occurs when there is an imbalance between free radicals and antioxidants, contributing to cellular damage, aging, and inflammation. The antioxidants in elderberries, particularly anthocyanins, help neutralize these free radicals, thus protecting the body’s cells, including skin cells, from damage [22]. In palliative care, where patients often experience chronic inflammation due to illness or treatments, elderberries’ anti-inflammatory properties can relieve pain, swelling, and discomfort, particularly for conditions such as arthritis, inflammatory skin diseases, and gastrointestinal inflammation.

#### 1.2.4. Skin Health and Healing

The skin-healing properties of elderberries make them especially relevant in palliative care, where patients frequently deal with compromised skin integrity [23]. The high levels of vitamin C in elderberries support collagen production, which is essential for skin regeneration and repair [24,25]. Collagen is a key protein in the skin that helps maintain its elasticity and strength. As such, elderberries are often used to treat dry, irritated, or aging skin [26]. In addition, the antioxidants in elderberries help prevent oxidative damage to the skin, which is essential for patients undergoing treatments like chemotherapy or radiation which can weaken the skin and make it more prone to irritation, dryness, and infections [16,25].

#### 1.2.5. Anti-Cancer Potential

Emerging research suggests that elderberries may have anti-cancer properties, primarily attributed to their high levels of anthocyanins and other antioxidants [12,27]. These compounds help protect cells from DNA damage and reduce the risk of tumor growth by combating oxidative stress and inflammation [19,28]. While elderberry’s role in cancer prevention requires further research, its antioxidant and anti-inflammatory effects may provide supportive care for cancer patients in palliative care, helping to improve their quality of life by addressing symptoms such as pain and fatigue associated with cancer treatment.

#### 1.2.6. Digestive Health

Elderberries also benefit digestive health thanks to their mild laxative effects [29]. In traditional medicine, elderberries have been used to relieve constipation and promote regular bowel movements [12]. Elderberries can offer gentle support to the digestive system for palliative care patients, especially those experiencing digestive discomfort due to medications or decreased mobility. The anti-inflammatory properties of elderberries may also benefit patients suffering from conditions like irritable bowel syndrome or inflammatory bowel disease.

Elderberry hydrolate, a product derived from the distillation of elderberry flowers or berries, contains many of the plant’s beneficial compounds in a gentle, water-soluble form. This makes it a suitable candidate for topical applications, especially for patients in palliative care who may experience sensitive skin or cannot tolerate more potent chemical-based products. Despite elderberry’s traditional use in skincare and its promising properties, exploring elderberry hydrolate within palliative care remains limited [30,31].

This review aims to fill this gap by examining the properties of elderberry hydrolate, focusing on its potential applications and benefits for patients in palliative care. By synthesizing existing research, we will explore elderberry’s antioxidant, anti-inflammatory, antimicrobial, and skin-soothing effects and how these properties may improve the well-being of palliative care patients. Furthermore, we will discuss the safety, efficacy, and practical considerations of incorporating elderberry hydrolate into palliative care regimens. Ultimately, this article highlights the therapeutic potential of elderberry hydrolate as a natural, supportive therapy for enhancing the comfort and quality of life of those facing serious health challenges.

## 2. Research Method

This review examines the properties of elderberry hydrolate (*Sambucus nigra*) and its potential applications in palliative care. For this purpose, the search was performed in the following online databases: b-on (https://www.b-on.pt/, accessed on 1 November 2024), PubMed (http://www.ncbi.nlm.nih.gov/pubmed, accessed on 1 November 2024), Web of Science (https://webofknowledge.com/, accessed on 1 November 2024), and Scopus (https://www.scopus.com/, accessed on 1 November 2024). The inclusion criteria comprised in vitro, animal, or clinical studies that investigated the chemical composition, biological activities, or therapeutic applications of elderberry hydrolate or its bioactive components. Studies exploring hydrolates or plant-based solutions relevant to palliative care were also considered.

The search terms included combinations such as “elderberry”, “*Sambucus nigra*”, “hydrolate”, “plant extract”, “antioxidant”, “anti-inflammatory”, “skin hydration”, and “palliative care”. The titles and abstracts of the identified articles were screened for relevance, and duplicates were excluded. No restrictions on publication year were applied to ensure a comprehensive review of the available evidence.

## 3. Elderberry Hydrolate

Elderberry hydrolate is obtained through steam distillation, which involves using steam to extract the essential oils and water-soluble compounds from the flowers or berries of the elderberry plant. During this process, the steam causes the plant’s volatile oils to evaporate, which are then condensed back into liquid form [32]. The aqueous distillate which remains after steam distillation and the separation of the essential oil whenever possible, known as the hydrolate, retains various bioactive compounds, including flavonoids, phenolic acids, and other water-soluble molecules that contribute to its therapeutic properties [33]. Unlike essential oils, which are highly concentrated and require careful handling, hydrolates offer a gentler alternative, making them more suitable for topical applications, especially in sensitive skincare for palliative care patients [34]. Hydrolates can be applied directly as a spray, compress, or incorporated into a cream; further research is needed to determine the most effective and practical application methods in palliative skincare [35,36].

### Chemical Composition

**Figure 1:**
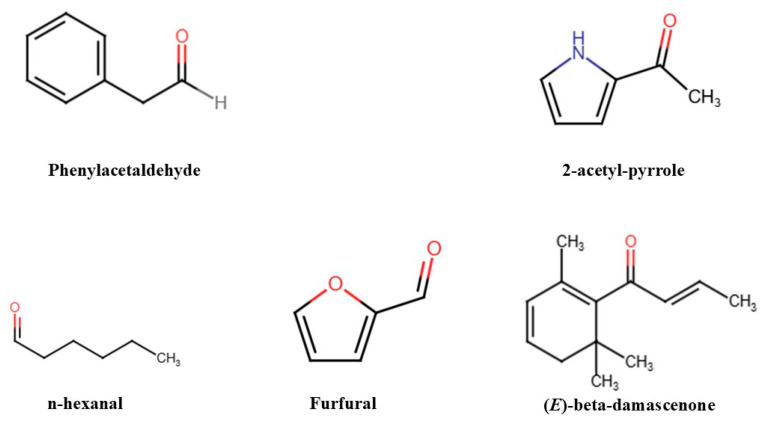
Chemical structure of compounds isolated from elderberry hydrolate, with pharmacological activity. Atoms are color-coded as follows: hidrogen (grey), nitrogen (blue), and oxygen (red).

Direct hydrolate analysis, i.e., analysis of the unprocessed hydrolate, revealed the presence of phenylacetaldehyde, 2-acetyl-pyrrole, n-hexanal, furfural, and (*E*)-beta-damascenone (Figure 1) [19]. Each of these molecules plays a role in the hydrolate’s overall effects on the skin and its potential uses in palliative care. Table 1 shows an overview of these key compounds and their respective benefits. All chemical structures were drawn using the RCSB PDB Chemical Sketch Tool (www.rcsb.org, accessed 4 February 2025) [37].

## 4. Discussion

The potential therapeutic value of elderberry hydrolate in palliative care lies in the diverse bioactive compounds it contains. The compounds identified in elderberry hydrolate—phenylacetaldehyde, 2-acetyl-pyrrole, n-hexanal, furfural, and (*E*)-beta-damascenone—play a critical role in its therapeutic potential, particularly in the context of palliative care. These bioactive compounds contribute to the hydrolate’s overall properties, benefiting palliative care patients, especially those experiencing compromised skin health.

For example, phenylacetaldehyde, with its mild antimicrobial and anti-inflammatory properties, could help soothe irritated skin and reduce inflammation, which is common in patients undergoing chemotherapy or other treatments that compromise skin integrity [61,62]. 2-acetyl-pyrrole, with its antioxidant properties, helps protect the skin from oxidative stress, which is essential for patients whose skin may be weakened or sensitive due to illness or medical treatments [63,64]. n-hexanal, possessing antimicrobial properties, could help prevent bacterial growth on fragile skin, thus reducing the risk of infections, a major concern for palliative care patients [65]. With its ability to reduce oxidative damage and promote skin healing, furfural could be particularly beneficial for patients with wounds or ulcers, promoting skin regeneration and reducing irritation [54]. Lastly, (*E*)-beta-damascenone, known for its antioxidant and skin-protective effects, could help prevent further damage to delicate skin, relieving dryness and maintaining skin health [60].

Together, these compounds enhance the overall skin-protective, anti-inflammatory, and healing properties of elderberry hydrolate, making it a valuable asset in palliative care. They improve patient comfort by addressing common skin concerns such as dryness, irritation, and inflammation and help maintain the skin’s integrity, which is often compromised in patients with advanced illnesses.

Elderberry hydrolate may exert its antioxidant effects through multiple biochemical pathways. One of the primary mechanisms is the direct scavenging of reactive oxygen species (ROS), whereby the volatile compounds in the hydrolate neutralize free radicals, reducing oxidative damage to skin cells [66,67]. Additionally, bioactive molecules such as furfural inhibit lipid peroxidation, preventing the oxidative degradation of skin lipids and thereby maintaining membrane integrity [68,69]. Another significant mechanism involves metal ion chelation, in which certain hydrolate compounds bind transition metals, reducing their ability to generate oxidative radicals that can exacerbate cellular damage [70,71].

Beyond direct antioxidant activity, elderberry hydrolate enhances the body’s natural antioxidant defenses by modulating endogenous enzymatic activity. Specific compounds within the hydrolate have been shown to increase the activity of key antioxidant enzymes, including superoxide dismutase (SOD) and glutathione peroxidase (GPx), which are crucial in neutralizing oxidative stress [72,73,74]. By stabilizing cellular redox balance, the hydrolate may help mitigate stress-induced skin degradation, making it particularly beneficial in palliative care, where skin integrity is often compromised.

Furthermore, elderberry hydrolate may influence several oxidative stress-related signaling pathways. Some of its bioactive components may enhance the activation of the Nrf2/Keap1 pathway, leading to the increased expression of antioxidant defense enzymes [75,76]. This upregulation strengthens cellular resilience against oxidative damage. Additionally, the hydrolate may inhibit NF-κB activation, thereby reducing inflammation-related oxidative stress, which is particularly relevant for individuals experiencing chronic inflammatory skin conditions [77,78,79].

The antioxidant mechanisms of elderberry hydrolate make it a promising adjunct in palliative skincare. By neutralizing oxidative stress, inhibiting lipid peroxidation, and modulating cellular antioxidant responses, elderberry hydrolate supports skin health and overall well-being.

### 4.1. Barriers to Implementation

While elderberry hydrolate presents significant therapeutic potential for palliative care, several practical and systemic barriers could impede its integration into clinical practice.

One major challenge is cost and scalability. Elderberry hydrolate production requires quality raw materials and specific distillation methods to ensure consistency and efficacy. These processes, mainly if performed on a small scale, may result in higher costs than more readily available synthetic alternatives. Additionally, ensuring an affordable supply chain without compromising quality remains a critical concern, particularly in underfunded palliative care settings.

Accessibility is another consideration. Elderberry plants, while common in certain regions, may not be readily available worldwide. This geographic limitation could restrict the widespread production and distribution of elderberry hydrolate. Furthermore, variations in plant quality due to environmental factors might affect the consistency of the final product, making its use less predictable in clinical settings.

Patient-specific factors also need to be considered. For instance, individuals with severe allergies or hypersensitivities may experience adverse reactions, even to natural products like hydrolates. Detailed safety profiling and patch testing are essential to minimize risks. Additionally, some patients or caregivers may prefer more familiar or conventional treatments, creating a barrier to adopting new therapies.

Lastly, regulatory challenges could delay or complicate the integration of elderberry hydrolate into standard care protocols. As a plant-derived product, hydrolates often fall into regulatory gray areas, which may necessitate additional testing to meet clinical standards for safety and efficacy. These hurdles could discourage healthcare providers from recommending its use, particularly in formal palliative care settings.

### 4.2. Research Gap

Despite the promising therapeutic properties of elderberry hydrolate, there is a significant lack of direct studies evaluating its application in palliative care [80]. This gap can be attributed to several interconnected factors. Hydrolates, although rich in bioactive compounds, are often overlooked in favor of more potent plant-based products like essential oils, which receive greater attention in research and clinical applications [81]. Additionally, dermatological care in palliative settings is frequently underprioritized compared to interventions focused on systemic conditions or pain management [2]. This imbalance in research focus may stem from a perception that skincare, while necessary, does not directly address life-threatening symptoms.

The complexity of hydrolates as therapeutic agents further compounds this issue. Variability in their composition, influenced by factors such as plant source and distillation method, creates challenges in standardization and validation. Moreover, palliative care research often encounters resource constraints, with funding and attention directed toward therapies perceived to have broader or more immediate impacts on patient outcomes. These challenges collectively contribute to the limited exploration of elderberry hydrolate in palliative care.

### 4.3. Future Directions

Addressing this research gap requires a multidimensional approach. The standardized chemical profiling of elderberry hydrolate is essential to ensure consistency and reproducibility in its therapeutic application. Establishing a well-defined chemical fingerprint can be a foundation for preclinical studies to evaluate its effects on common palliative dermatological issues such as pruritus, irritation, and wound healing.

Clinical studies should prioritize assessing the safety, efficacy, and acceptability of elderberry hydrolate among palliative care patients. Small-scale trials could provide valuable insights into its impact on skin health and overall quality of life.

Comparative effectiveness research could further contextualize its benefits by juxtaposing its outcomes with those of existing topical treatments. In addition to biochemical and clinical assessments, Patient-Reported Outcome Measures (PROMs) play a crucial role in evaluating the efficacy of interventions in palliative care. Given the highly subjective nature of dermatological symptoms such as pruritus, dryness, and discomfort, PROMs can provide valuable insights into patient perceptions of symptom relief and overall well-being. Incorporating PROMs into future studies on elderberry hydrolate could help quantify its impact on quality of life, patient satisfaction, and treatment adherence. Moreover, these measures would complement objective biomarkers by capturing aspects of care that are most meaningful to patients, such as improved comfort and reduced irritation. By integrating PROMs, researchers can better assess the real-world applicability of elderberry hydrolate as a supportive therapy in palliative care.

Interdisciplinary collaboration between dermatologists, palliative care specialists, and pharmacologists is crucial to designing research that aligns with clinical needs while addressing practical considerations. Additionally, incorporating patient and caregiver perspectives through qualitative studies can ensure that elderberry hydrolate is developed and implemented in a manner that aligns with the lived experiences of those in palliative care.

By addressing these avenues, future research can pave the way for integrating elderberry hydrolate into evidence-based palliative care practices, potentially offering a natural and gentle alternative for improving patient comfort and well-being.

## 5. Conclusions

Elderberry hydrolate represents a promising natural solution for addressing dermatological challenges in palliative care. Its demonstrated anti-inflammatory, antioxidant, and skin-healing properties offer a gentle yet effective option for improving patients’ quality of life with compromised skin health. By alleviating common symptoms such as pruritus, irritation, and dryness, elderberry hydrolate could significantly enhance patient comfort and dignity in their final stages of life. Integrating elderberry hydrolate into routine palliative care can address dermatological concerns and improve patients’ holistic well-being. By bridging traditional botanical knowledge with contemporary clinical practices, this natural therapy can redefine care standards.

However, the potential of elderberry hydrolate in palliative care remains underexplored. This review has highlighted the need for further research to validate its efficacy and safety, including standardized chemical profiling, preclinical studies, clinical trials, and PROMs. Given the highly individualized and subjective nature of skin-related symptoms in palliative care, PROMs will be essential for assessing patient experiences, comfort, and the perceived benefits of hydrolate-based treatments. Addressing practical barriers such as cost, accessibility, and regulatory challenges will be crucial for integrating this therapy into routine palliative care practices.

Despite these challenges, the therapeutic promise of elderberry hydrolate underscores its value as a complementary therapy. Future research and interdisciplinary collaboration can pave the way for its broader application, offering palliative care patients a natural, non-invasive alternative to enhance their well-being and comfort. By bridging the gap between traditional knowledge and evidence-based practices, elderberry hydrolate can become an integral part of holistic care strategies tailored to the unique needs of palliative patients.

## Figures and Tables

**Table 1:** Characterization of key compounds found in elderberry hydrolate and their potential benefits.

Compound	Characterization	References
Phenylacetaldehyde	An aromatic aldehyde compound known for its sweet, floral, and honey-like scent. It is commonly found in various essential oils and hydrolates, contributing to the overall fragrance profile. It has mild antimicrobial and anti-inflammatory properties, which could help soothe irritated skin and reduce inflammation.	[38,39,40,41]
2-acetyl-pyrrole	A heterocyclic compound known for its characteristic nutty, roasted aroma. It is identified in several plant hydrolates and essential oils and is noted for its antioxidant properties. Antioxidants help neutralize free radicals, protecting the skin from oxidative stress and premature aging.	[42,43,44,45]
n-hexanal	An aliphatic aldehyde with a grassy, slightly green odor, and it is often associated with the scent of freshly cut grass. It possesses antimicrobial properties, potentially helping to prevent bacterial growth on the skin.	[46,47,48]
Furfural	A volatile organic compound with a characteristic odor of almonds or burnt sugar. A product of the breakdown of plant materials, particularly sugars, known for its antioxidant and anti-inflammatory effects. It may play a role in reducing oxidative damage to the skin, thus supporting skin health and alleviating irritation or redness. It could also contribute to the hydrolate’s ability to promote skin healing.	[49,50,51,52,53,54,55]
(*E*)-beta-damascenone	A volatile compound commonly found in the aroma profiles of many plants, known for its floral, fruity scent reminiscent of roses. This compound has been studied for its antioxidant properties, which contribute to reducing free radical damage to the skin. It has potential as a skin-protective agent.	[56,57,58,59,60]

## Data Availability

Data supporting the findings and conclusions are available upon request from the corresponding author.
